# A relational turn in climate change adaptation: Evidence from urban nature-based solutions

**DOI:** 10.1007/s13280-024-02090-9

**Published:** 2024-12-07

**Authors:** Sean Goodwin, Marta Olazabal, Antonio J. Castro, Unai Pascual

**Affiliations:** 1 Basque Centre for Climate Change (BC3), Edificio Sede 1, 1º Planta, Parque Cientifico UPV/EHU B/Sarriena s/n, 48940 Leioa, Bizkaia Spain; 2 University of Almería, Almería, Spain; 3 Basque Foundation for Science, Ikerbasque, Bilbao, Spain; 4 Department of Biology and Geology, Andalusian Centre for Global Change - Hermelindo Castro, University of Almeria, Ctra. Sacramento s/n, 04120 Almería, Spain; 5 Centre for Development and Environment, University of Bern, Bern, Switzerland

**Keywords:** Adaptation success, Cities, Climate change adaptation, Imaginaries, Relationality, Urban nature-based solutions

## Abstract

The emergence of nature-based solutions (NbS) in science, policy, and practice signals a paradigmatic shift in urban climate change adaptation, yet empirical investigations into its impact on adaptation definitions and progress tracking remain scarce. Addressing this gap, we conducted thematic analysis on semi-structured interviews (*n* = 15) with practitioners responsible for implementing and evaluating urban NbS in different countries. We provide a nuanced understanding of urban adaptation goals within urban NbS according to the insights from these practitioners, extending beyond hazard mitigation and towards cultivating and strengthening relationships between humans and nature. Tracking adaptation progress towards such relational adaptation goals requires acknowledging knowledge pluralism and the diversity of human–nature relations. We propose an alternative definition of adaptation supported by our data that aims to foster a more holistic approach to urban climate adaptation that accounts for the potential benefits of urban NbS across interconnected climate, biodiversity, and social goals.

**Supplementary Information:**

The online version contains supplementary material available at 10.1007/s13280-024-02090-9.

## Introduction

The emergence of nature-based solutions (NbS) has contributed to a shift in how climate change adaptation success in cities is understood and what information and tools are needed to track its progress (Raymond et al. 2017). It is increasingly assumed that urban adaptation progress is linked to “greening” cities (Depietri and McPhearson 2017; Angelo 2019; Dorst et al. 2019). The rise of the NbS agenda is currently influencing narratives on what successful adaptation looks like in cities in this way (Melanidis and Hagerman 2022; Neidig et al. 2022; Westman and Castán Broto 2022). However, critics highlight how the narrative shift has not necessarily clarified how to recognise or evaluate successful adaptation in practice (Tozer et al. 2020; Woroniecki et al. 2020; Westman and Castán Broto 2022). This is partly because adaptation projects often suffer from poorly articulated definitions and expectations about their success (Olazabal et al. 2019). In addition, they are rarely (if ever) followed up on (Eriksen et al. 2021; Mills-Novoa 2023).

Conceptually, adaptation is not safe from ambiguities or rigidities either. Critical scholars highlight the need to challenge received wisdom embodied within dominant framings of adaptation (Castán Broto et al. 2024; Olazabal et al. 2024). Those include definitions from the Intergovernmental Panel on Climate Change (IPCC) and are argued to limit the ontology and epistemology of adaptation to an overly technocratic and universalised process of “adjustment” to changing biophysical conditions (Goldman et al. 2018; Dujardin 2020; Mabon et al. 2022). In its ontology, adaptation is argued to additionally be a function of social vulnerability and resilience that depends on the strength of human–nature relationships and (inter)subjective felt experiences of climate impacts (Goldman et al. 2018; West et al. 2020; Nightingale et al. 2020; Haverkamp 2021; Olazabal et al. 2021). Taking into account these components of adaptation requires an epistemology within the adaptation process (encompassing the design, implementation and implementation of adaptation plans, strategies, and interventions) that includes pluralistic and transdisciplinary forms of (local) knowledge (Rahman et al. 2023; Kythreotis et al. 2024) that have been historically marginalised in dominant adaptation discourses over scientific and technical forms of knowledge (Fischer et al. 2012; Chmutina et al. 2023; Wise et al. 2014).

Dominant framings of adaptation could be enriched by what are termed “urban climate imaginaries,” defined as “sets of ideas about what the world is and how it works” (Lawhon et al. 2023, p. 128) shaped by individual and collective visions of cities (Westman and Castán Broto 2022; Castán Broto et al. 2024). When employed as a theoretical frame to interpret local understandings and evaluations of adaptation success, these imaginaries have the potential to enrich real-life adaptation processes by generating “common understandings of important issues, underlying causes, and pathways toward optimistic futures” (Cork et al. 2023). However, there has been little empirical use of the concept of climate imaginaries applied to urban NbS in attempting to challenge dominant framings of (urban) adaptation (Olazabal et al. 2024), with recent research suggesting instead that adaptation planning has become increasingly homogenised (and globalised) over time (Westman and Castán Broto 2022; Westman et al. 2023). Analysing local urban climate imaginaries is therefore critical and timely (Nalau and Cobb 2022; Castán Broto et al. 2024; Pelling et al. 2024). Urban NbS make a particularly interesting application of the concept of urban imaginaries because of the unique entry point of urban NbS to adaptation that centres the importance of highly context-dependent human–nature relationships that contradict technocratic narratives on adaptation (Dorst et al. 2019; Pörtner et al. 2023; Rochell et al. 2024).^1^

To fill this gap, and thus, understand how imaginaries, goals, and their evaluation connect with framing urban NbS to climate adaptation, we ask two interconnected research questions: (1) What does adaptation success mean in the context of urban NbS according to local NbS practitioners, and (2) whose and what types of knowledge are important for developing their definitions and assessing progress towards adaptation goals?

We answer these two questions based on insights gained from practitioners responsible for adaptation processes for urban NbS on the ground. The urban climate imaginaries emerged from a thematic analysis of targeted interviews conducted with 14 concluded urban NbS projects around the world (*n* = 15 interviews) associated with a diversity of urban geographies, actors behind NbS processes, and adaptation goals. We performed a qualitative (thematic) analysis based on information from in-depth interviews since eliciting and analysing urban climate imaginaries require deep insight into individual and collective worldviews (Verlie 2019; Westman and Castán Broto 2020; Iossifidis and Garforth 2022). The goal of this thematic analysis is to explore a research area that has not been widely studied and does not have much existing data to rely on. We are not trying to make broad generalisations based on our interview results, but instead, we aim to help fill this gap by contributing to "a situated, reflexive and theoretically embedded practice of knowledge generation or construction, rather than discovery" (Braun and Clarke 2021) (see Supplementary Information S1 for further discussion).

## Theoretical framework to understand urban climate imaginaries

The concept of urban climate imaginaries provides a useful body of theory to make underlying ontological and epistemological assumptions explicit within local framings and evaluations of adaptation success through NbS (Westman and Castán Broto 2020; Cork et al. 2023). Urban climate imaginaries come with their own vision of what successful adaptation means (or “ways of being” adapted to climate change, i.e. ontology) and how to recognise adaptation success in practice (or “ways of knowing” adaptation is occurring, i.e. epistemology) (Lawhon et al. 2023). Imaginaries on ways of being and knowing adaptation converge to form the processes that operationalise desired states of being and the knowledge used to verify them, referred to as “ways of doing” (Goldman et al. 2018).

Imaginaries of ways of being adapted to climate change include any conceptualisation of what adaptation is, as well as what success in adaptation may look like. They can manifest explicitly, for example in institutional definitions of adaptation like those of the IPCC (Eriksen et al. 2015; IPCC 2023a), or may implicitly underpin how adaptation success is defined and evaluated, for example through heuristics (Preston et al. 2015; Vogel and Henstra 2015). Heuristics of adaptation include ontological assumptions of what adaptation is and what its practice requires by those designing, implementing, studying, or benefiting from it, for example that is it “local,” “novel,” and “urgent” (Nalau et al. 2021). Other imaginaries are more normative in that they contain assumptions about what adaptation should be in order to succeed. These include, for example, that adaptation processes must account for affective, intersubjective felt experiences on the understanding that climate change impacts are affective in nature (Nightingale et al. 2022), or that they should be participatory and encourage learning processes (Nalau et al. 2021). Ontological assumptions about adaptation are also influenced by other concepts, for example what it means to be vulnerable or resilient to climate change (Holling 1973; Folke et al. 2010; IPCC 2023b).

Ways of knowing adaptation describe imaginaries of what and whose knowledge counts as valid, legitimate, and useful to define and evaluate adaptation success. These imaginaries have implications in how they define what kind of knowledge goes into defining and evaluating adaptation and whose voice counts within those discussions that perpetuate assumptions about valid, legitimate, and useful knowledge (Eriksen et al. 2015; Chmutina et al. 2023). Ways of knowing adaptation operate in an individual and collective manner in climate change governance, and are also therefore a function of “discursive” power in terms of who gets to decide on definitions and which voices are included within decision-making processes (Woroniecki et al. 2020; Westman and Castán Broto 2022; Arias-Arévalo et al. 2023). Discursive power dynamics do not only operate on the intergovernmental level, but filter down too into local practices of citizen participation in adaptation processes. Different city-level adaptation policies, plans, and interventions embody their own epistemology on whose voice will be heard and how that influences decision-making processes, and those left out (Olazabal et al. 2021).

Ways of being and knowing urban adaptation converge in the processes that bring them to life (ways of doing). Beyond taking an NbS approach, ways of doing urban adaptation extend to broader practices and procedures used within adaptation processes, for example through the use of different theories and approaches to monitoring, evaluation, reporting, and learning (MERL) (Eriksen et al. 2021). In particular, theory of change approaches are advocated for in adaptation projects within MERL processes, and in particular for NbS generally whether or not they aim to have adaptive benefits (IUCN 2020). Using this approach often relies on back-casting or visioning processes. In addition, high-level goals of adaptation tend to be defined in generic terms (e.g. “increase climate resilience”) and traced back to the present moment, identifying the actions and resources required to bring them about and identifying what information is needed to assess progress towards meeting adaptation goals (Ssekamatte 2018; Leiter 2021).

Theorising the local practice of adaptation through the lens of imaginaries of ways of being and knowing that converge in ways of doing helps identify key weaknesses in current adaptation practice as well as opportunities for disrupting hegemonic imaginaries (Castán Broto et al. 2024). For example, given that adaptation approaches like NbS are highly context-dependent to the local scale (Dorst et al. 2019; Pörtner et al. 2023), limited ways of being (e.g. unclear adaptation goals) and knowing (e.g. lack of inclusive processes for developing them) may result in similarly stunted or potentially harmful ways of doing adaptation (i.e. unintended or maladaptive outcomes) which ultimately stifle adaptation progress (Goldman et al. 2018; Nightingale et al. 2020; Mabon et al. 2022).

## Materials and methods

### Data collection through interviews

Key informants were identified from a global sample of urban NbS for adaptation (see Goodwin et al. (2023)). Purposive sampling of NbS was done in a two-step process. First, an initial sample of NbS was selected that first evidenced some form of MERL processes (*n* = 74) to ensure informants were able to reflect on their experience with the NbS before, during, and after implementation. A sub-sample (*n* = 15) (See Table S1 for full sample) was then identified based on additional purposive and strategic criteria accounting for (1) the experience of the informant with the development of the NbS they were engaged with (verified through asking whether they formed part of the core design/implementation team of the NbS), (2) diversity of NbS (in the initial sample, mostly categorised as wetlands, public parks/gardens, green roofs, urban forests, river re-naturalisation, and urban agriculture as per Goodwin et al. (2023)), and (3) geographical diversity (relating to the five primary world regions according to the United Nations categorisation—Africa, Americas, Asia, Europe, and Oceania) (United Nations 1999). The availability and ability of potential informants to take part in online interviews in English within the interview period (March to May 2023) further acted as a sample limitation criterion (see Table S2 for the effect this had on the sample). Regarding (2) and (3), diversity relates to the diversity of NbS types and geographies present within the initial sample (*n* = 74).

Our aim within this study is to describe the themes we found in our data that we believe tell a “rich, complex, and multifaceted story about the patternings of [our] phenomena of interest” (*ways of being*, *knowing,* and *doing* urban adaptation through NbS) based on the situated knowledge(s) of informants (Sim et al. 2018). There is some debate about what sample size is enough to achieve a detailed description, but as a general guideline, some authors suggest that a sample of six to sixteen participants may be enough. However, the size of the sample alone is not a reliable way to measure whether the themes in the data are fully explored. Instead, in addition to having a sample size in or above this range, answering several reflective questions from Braun and Clarke (2021) can give more credibility to thematic analyses. These questions include the type of thematic analysis being used, the underlying assumptions of the research (such as its paradigmatic, ontological, and epistemological foundations), how codes and themes are defined, and how any numeric criteria for evaluating samples are justified.

With this in mind, our sample of 15 fits within the suggested range of six to sixteen. However, we also understand that saturation is not about reaching a specific number of interviews but rather ensuring that the sample size is sufficient to answer the research questions. The interviews were conducted in a semi-structured and in-depth way, which allowed us to support the themes with quotes from the participants and thereby provide detailed answers to our questions. As mentioned in the “Introduction”, our research takes a constructivist approach, where knowledge and meaning are not discovered but are interpreted. This interpretation relates to the situated knowledge of informants and their worlds, rather than on a general level. This interpretive process was made systematic through our coding system, as described in Sect. "Thematic analysis of interview data". While the approach was exploratory, it was not arbitrary, and we aimed to stay true to the words of our participants (see Supplementary Information S1 for further discussion). See especially Table S3.

Two questions directed the semi-structured interviews. First, informants were asked about the process of selecting information to evaluate their urban NbS that they were closely involved with, which allowed us to undertake detailed discussion not only on MERL processes but also on the overall NbS aims and how the informants understood adaptation on the ground. Second, informants were then asked to reflect on the overall process of MERL they were involved in from their own perspective (see Supplementary Information for the list of informants and inter- 304 view guide). See especially Table S4 for more detailed questions and probes.

### Thematic analysis of interview data

**Fig. 1:**
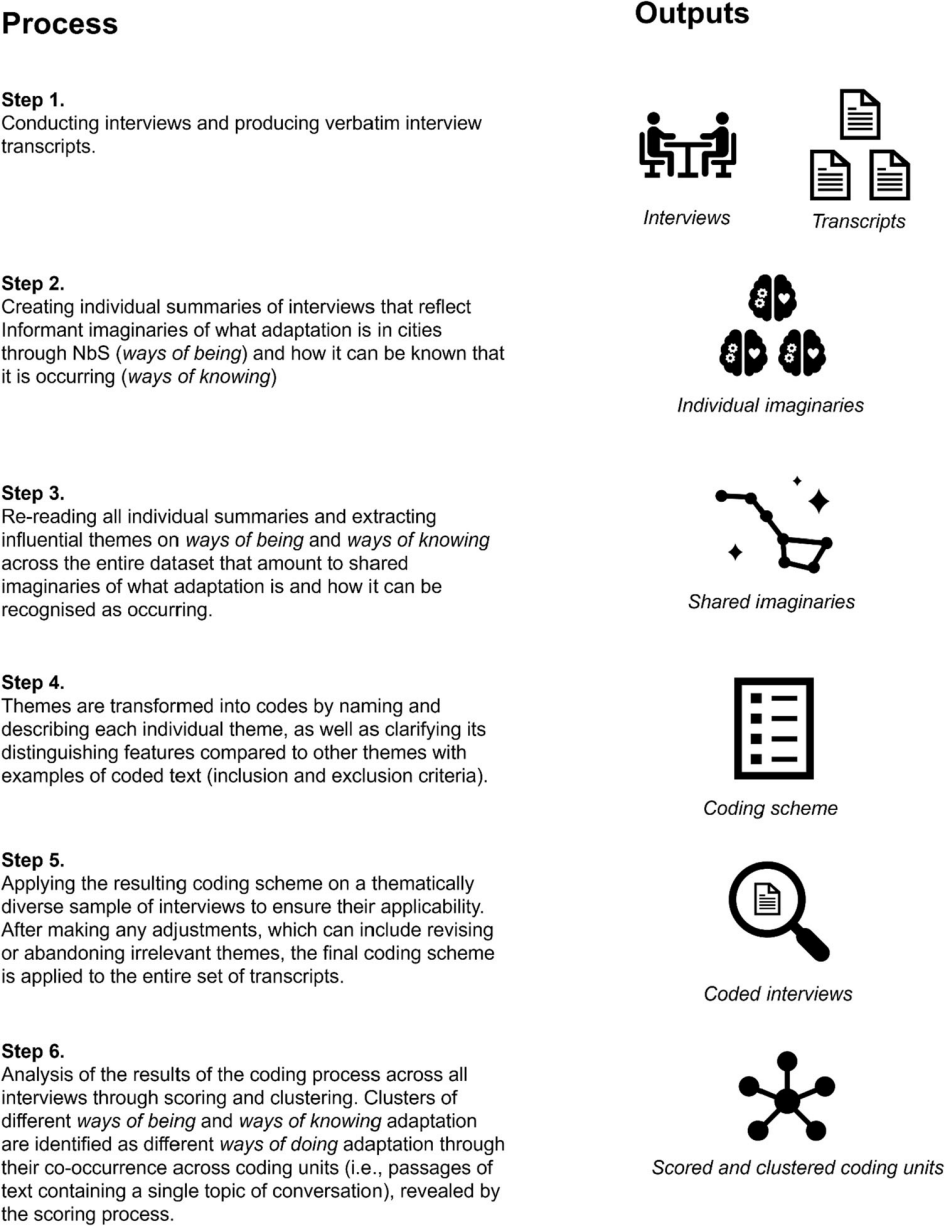
Summary of the thematic analysis process

Interviews were transcribed verbatim and analysed following the approach to thematic analysis suggested by Boyatzis (2010) using NVivo software (release 1.2.1). Following this approach, short individual summaries (3–4 sentences) were produced for each interview regarding both *ways of being* and *ways of knowing*. A meta-summary was then produced that drew together the most influential themes across the individual summaries. We deemed themes to be influential (Braun and Clarke 2006) if they played an influential role in how the informant defined adaptation and what information was needed to evaluate progress towards adaptation goals. These themes were then transformed into codes, which in turn were applied to all interviews (Fig. 1). The answers given by informants were broken down into coding units which were each coded to one or more theme. Each coding unit was a separated passage of text that contained a single topic of conversation. Each interview transcript then became the unit of analysis.

A hybrid approach was taken to the thematic analysis that was part inductive and deductive. We deductively used the concept of urban climate imaginaries as a way to locate more abstract theories of ways of being and knowing urban adaptation through NbS within the experiences and perspectives of informants throughout the thematic analysis process. The individual summaries aimed to inductively encapsulate the perspectives and worldviews (or imaginaries) of each informant in terms of how they understood what adaptation is (ways of being) and how it can be recognised (ways of knowing). Then, the meta-summary reflected the shared imaginaries on ways of being and ways of knowing that were later translated into individual codes that particularise the most important features of those imaginaries across the data. In this way, the concept of imaginaries was used to frame the individual interview responses to uncover ways of being and ways of doing in the data in the form of themes.

The first result of the thematic analysis involved the themes themselves which are summarised descriptively. The second result was then a detailed analysis of the themes through a process of theme scoring and clustering (Boyatzis 2010). This involved running matrix queries in NVivo to determine the magnitude of the co-occurrence of themes (called “scoring”) across all coded passages of text (coding units). The resulting co-occurrence matrix was analysed to identify which themes co-occurred often, which was checked against a qualitative analysis of the coding units, which suggest they may be thematically grouped (called “clustering”). The different thematic clusters of ways of being and ways of knowing urban adaptation through NbS were then taken as ways of doing adaptation.

The differences between distinct ways of doing adaptation that resulted from the clustering were additionally explored through an ANOVA-type nonparametric multivariate inference test using the npmv package in R version 4.3.2 (Burchett et al. 2017). This test is a useful nonparametric statistical method to verify differences between two groups within the data where there are numerous binary response variables (i.e. co-occurrence of numerous themes within the same coding unit) with low sample sizes and high-dimensional data as is the case here (Van Valkengoed et al. 2021; Fouani et al. 2024).

## Results

### Theme descriptions

All informants partly conceptualised adaptation as a process of protecting communities against climate hazards, with some additionally noting that it was also a process of building relationships among people and/or the natural world (Table 1). We called these themes “hazard-centric” and “relationship-centric,” respectively. Relatedly, informants spoke of adaptation as either something interconnected or separate between human communities and ecosystems. Distinct from how informants conceptualised adaptation, the theme on *adaptation success* then summarised what the personal (though shared across the sample) accounts of what informants felt were key attributes of their NbS (i.e. of different processes of design and implementation) that made it successful in providing adaptive and associated co-benefits. Though it is not possible to exclude that these attributes could apply more broadly to the successful implementation of NbS, discussions focused on those factors noted to be particularly relevant to the intersection of NbS and adaptation.

**Table 1:** Definitions of *ways of being* adapted to climate change according to informants (*n* = the number of interviews the theme appeared in). All theme and sub-theme names are later italicised in the main text to signal that the term used is reference to the coded themes, as these terms may be used differently in other contexts

Guiding theory	Themes	Sub-themes	Description	Quote (examples)
*Ways of being*	Hazard-centric (*n* = 15)	Reducing climate impacts (*n* = 15)	Protecting against the impacts of climate hazards by reducing exposure or sensitivity to their effects.	“[Being adapted to climate change] means reduc[ing] the urban heat island effect we are suffering from” (Informant #3)
		Supporting adaptive capacity (*n* = 5)	Preparing populations to cope in the face of climate hazards.	“We also found that the level of awareness was quite low, so some people didn't even know what climate change was or meant. So we then decided that in order to raise awareness, it's not enough to just have awareness sessions to tell people what climate change is.” (Informant #4)
	Relationship-centric (*n* = 10)	Among people (*n* = 7)	Adaptation is achieved by building relationships among people within society. Creating and strengthening relationships of care within communities create opportunities for mutual assistance and support to prepare for, and survive, climate hazards.	“[Adaptation is achieved by] doing the heart to heart, [developing] people connections, … trust and human bonds” (Informant #2)
		With nature (*n* = 6)	Adaptation is achieved by building relationships between people and nature. The lack of mutually beneficial and supportive relationships between humans and nature is highlighted as a key driver of both climate change and ecosystem decline. Creating and reinforcing these relationships is then crucial to addressing their underlying drivers.	“… there is this movement of people working towards bringing and raising consciousness about the importance of having access to the waters again. And getting the words of the rivers out. So, the idea is more like being the speaker for the rivers” (Informant #13)
		Both humans and nature (*n* = 4)	Adaptation is achieved by both building relationships among people within society and between people and nature.	“… [people] feel that this is their belonging… this plant belongs to me and this plant needs to grow. And that's why this land needs to be very secured and safe. And that way, partnership is built and ownership is also built because of that.” (Informant #11)
	Connectivity (*n* = 12)	Interconnected (*n* = 10)	Adaptation is something deeply interconnected between humans and the natural world.	“[Defining] nature-based solutions as something that used nature to simultaneously help nature and the social aspects…. splitting it down into sub-compartments helped people see how interconnected everything is” (Informant #7)
		Separate (*n* = 2)	Adaptation in human and natural systems are separate.	“It's a garden… [the NbS] will recover…. they could sort of handle those climate impacts. But then the other thing is what to do when there's drought, and [the farmers] have to adapt their cultivation technique.” (Informant #12)
	Adaptation success (*thriving*) (*n* = 14)	Context sensitivity (*n* = 9)	For an NbS to thrive, it needs to respond to the social and ecological context it exists within.	“…the whole entire context was something that was very favourable to these other groups growing.” (Informant #14)
		Flexibility (*n* = 4)	For an NbS to thrive, it must be implemented and evaluated in ways that enable reflection, learning, and path restructuring towards the intended goal.	“[We] need to be agile… and more flexible on the situation on the ground.” (Informant #5)
		Sustainability (*n* = 10)	For an NbS to thrive, it must persist over time, to continue and increase the provision of benefits into the future following implementation, and be “self-renewing” because it is closely integrated into how the city system functions.	“[NbS] interventions aren't supposed to finish with the end of the project, that's when they start. And so in order to see if you were successful at all or not, you have to go there, not at the end of the project when everybody is still around and the beneficiaries still remember you.” (Informant #10)
		Synergy (*n* = 9)	For an NbS to thrive, it must create and strengthen synergetic links between actors involved in implementing the project as well as beneficiaries. Synergy means that different actors are brought together to do something in a better way than if they had done so alone, as all involved bring to bear their unique skills, knowledge, experience, and other strengths to contribute to the project that fills in the gaps left by others.	“We actually worked in partnership with the airforce, the military police, Indigenous peoples, religious communities, African heritage religious communities, catholic communities, evangelical communities, you name it they were there, public agencies, federal police, the army… we had a lot of support. We got together whatever anyone could donate, bring it together. For example, the cavalry, they would bring the manure, so we composted it …we had to rotate the place, but it worked perfectly, it was a really rewarding experience.” (Informant #2)
		Growth (*n* = 7)	For an NbS to thrive, it must grow, meaning that the physical boundaries of the project expand either on the site or across the city, or that more people joined or benefited from the project over time.	“It's all to do with nature. How do you know that the species is successful? How is life successful? Ultimately when it breeds, when it spreads…” (Informant #2)

The main themes relating to *ways of knowing* urban adaptation through urban NbS centred around identifying from and for whom knowledge needed to be gathered and whether or not they expressed a belief in a hierarchy among these different forms of knowledge (Table 2).

**Table 2:** Types of knowledge used to identify when and how adaptation is taking place (*n* = the number of interviews the theme appeared in). All theme and sub-theme names are later italicised in the main text to signal that the term used is reference to the coded themes, as these terms may be used differently in other contexts

Guiding theory	Themes	Sub-themes	Description	Quote (examples)
*Ways of knowing*	Knowledge from (*n* = 14)	Local (*n* = 14)	Knowledge that is accumulated by people local to the project over time, which is sourced from their direct experiences, observations, interactions, and relationships with the site of the NbS.	“Feelings, […] perspectives, [and] lived experience […] of the people living there [close to the NbS]” (Informant #9)
		Scientific (*n* = 12)	Knowledge that is created following a scientific method that was “reproducible” and “unbiased.” Key to producing scientific knowledge was following set methodologies and protocols, especially relating to the natural sciences.	“[the information we use is good] because it is performed with a methodology, they are always done [in the same way], we are doing a transect of, whatever, 15 min, taking notes of all the birds we have heard.” (Informant #15)
		Technical (*n* = 13)	Knowledge that may have been produced using technology or technical expertise (for example, engineers, architects) in some way. For example, this includes using instruments or software to numerically evaluate the performance of an NbS in terms of its inputs, outputs, costs, or efficiency, which was not necessarily collected in a way that strictly follows a scientific methodology.	“The flooding officer, which is working downstream of [the NbS], says it's [the NbS] made a noticeable difference already to some of their projects.” (Informant #7)
		Indigenous * (*n* = 2)	Knowledge was framed to encompass the knowledge held by Indigenous peoples based on their worldviews, cosmologies, experiences, and relationships that are intimately tied to the lands and waters of the territory where an NbS is implemented.	Informant #10 noted the specific work and philosophies of Indigenous people informed their belief that “progress is something that needs to be re-evaluated… the new progress needs to look back,” in noting the importance of redefining people’s relationship with nature in urban environments
	Knowledge for (*n* = 15)	Human systems (*n* = 15)	Knowledge that is needed to understand adaptation dynamics within human systems.	Informant #5 specified that the goal of project evaluation was to know that “the capacity of participating local government authorities surrounding [the city] [had] improved their service delivery”
		Natural systems (*n* = 11)	Knowledge that is needed to understand adaptation dynamics within natural systems.	Informants noted the importance of creating knowledge to understand ecological benefits within natural systems, such as “diversity of species, structural diversity, and with the idea of it [the NbS] becoming a functional ecosystem.” (Informant #6)
		Both (*n* = 12)	Knowledge that is needed to understand adaptation dynamics that connect both human and natural systems.	“… we're doing [the NbS] because we want to have access to the rivers. Not only for ourselves, but for everyone and for the river in itself…” (Informant #13)
	Knowledge hierarchy (*n* = 14)	Hierarchy (*n* = 8)	There is a hierarchy among the different kinds of knowledge that may be used to understand adaptation.	In noting the differing levels of credibility of different kinds of knowledge for assessing NbS, one informant noted “…it's not science, it's more technical support… they publish things, but it isn't like in the university.” (Informant #15)
		Pluralism (*n* = 8)	There is no hierarchy among the different kinds of knowledge that may be used to understand adaptation, rather, all forms of knowledge are equally useful and valid.	“We have to put ourselves in a very humble position. It is not because we have the means or the knowledge that we can tell everyone how it's going to be. This is exactly the kind of thought that brought us to the crisis. This is a hierarchical, dialectic process. … This is limited. … This is not systemic, for me, we need a systemic solution.” (Informant #2)

*Note that because of the inductive method used, this is a synthesis of how Indigenous knowledge(s) was understood by participants, who did not specifically identify as Indigenous themselves. This cannot and should not be used as a broader definition of Indigenous knowledge(s). This applies more broadly to how other forms of knowledge were defined. These definitions should be understood as how the different forms of knowledge mentioned by informants were understood by them

### Scoring and clustering of themes

Scoring and clustering of themes on *ways of being* and *ways of knowing* are used here to distinguish *ways of doing* adaptation. These *ways of doing* are therefore the result of converging *ways of being* and *ways of knowing* urban NbS.

### Distinguishing between ways of doing urban adaptation through NbS

Upon an initial analysis of the co-occurrence of themes across coding units (i.e. thematic passages of text), the *hazard-centric* way of understanding adaptation seldom co-occurred with the *relationship-centric* understanding in interviews, leading us to explore the possibility that they are thematically distinct (see co-occurrence matrix in Table S5 in Supplementary Information). These two main ways of understanding adaptation only intersect in the data through the 'supporting adaptive capacity' sub-theme, which offers common ground between them.

The ability for these two themes to distinguish two groups within the data was further supported by the nonparametric multivariate inference test as it indicated a highly significant difference between those coding units coded with either theme (ANOVA test statistic = 13, df = 8, df2 = 927, *p* < 0.0001).

**Fig. 2:**
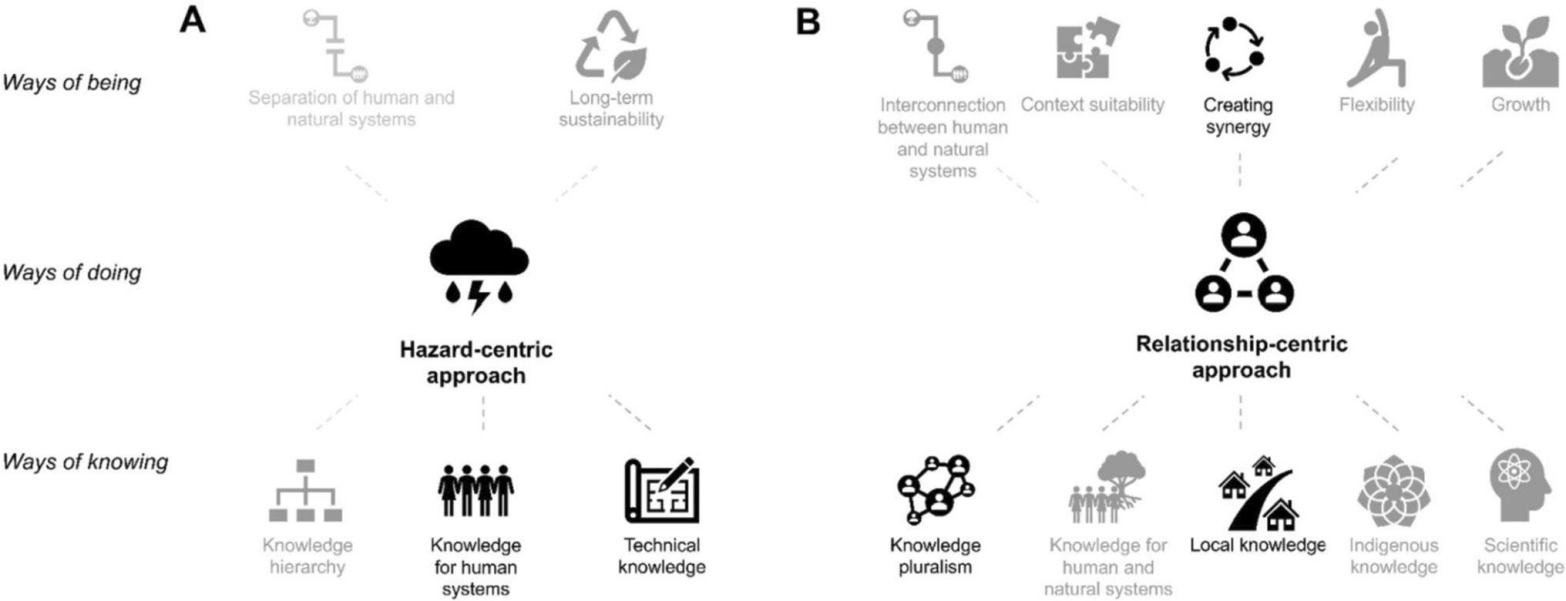
Summary of the scoring and clustering of themes into two groups: **A** the *hazard-centric* approach and **B** the *relationship-centric* approach. Themes were allocated to either group based on whether they more often co-occurred with one theme over another, where those with the higher statistically significant differences appear more prominently

This test additionally provides insight into defining features of each approach as it further provides information about the probability of a coding unit being coded as either approach in addition to another one of the themes on ways of being and knowing adaptation (Table S7, summarised in Fig. 2). The trends within these results match the absolute differences in co-occurrence of these two themes and all other themes (Table S6) and so they are discussed below together. As these are relative differences, it is difficult to identify a specific cut-off value that would identify which variables are the most statistically significant. However, presenting the results in this way is more easily interpretable because they are on a common scale. Taking a pragmatic approach, we discuss those sub-themes with the largest difference in probability per theme (Burchett et al. 2017).

In terms of ways of being*,* neither approach could be said to strongly relate to a s*eparation of human and natural systems* in terms of how adaptation was defined, though only co-occurred with the *hazard-centric approach* (Table S7). Both approaches were associated with an *interconnected definition of adaptation between human and natural systems*, though this was slightly more so within the *relationship-centric approach*. The largest difference in co-occurrence between understandings of adaptation success related to *creating synergy*, which more strongly related to the relationship-centric approach, along with weaker connections to *context suitability, flexibility*, and *growth*. Discussions of the importance of *sustainability* as a measure of success co-occurred more often with the hazard-centric approach.

Regarding ways of knowing, the largest differences in co-occurrence could be seen in stronger connections between the importance of *creating knowledge for human systems in isolation* with the *hazard-centric approach*, with more slight but notably higher association between the *relationship-centric approach* and *creating knowledge for human and natural systems in an interconnected way*. The *relationship-centric approach* was more strongly differentiated from the *hazard-centric approach* in its association with the importance of *local knowledge*, while the *hazard-centric approach* more often co-occurs with *technical knowledge*. The difference between associations between both approaches and the importance of *scientific* and *Indigenous knowledge* only showed slight differences. Neither approach co-occurred strongly with the belief in *knowledge hierarchy*; however, the *relationship-centric* approach was more explicitly related to the importance of *knowledge pluralism* compared with the *hazard-centric approach*.

To contextualise these results further, the hazard-centric approach was more often seen in interviews about NbS located in Africa relating to urban forests and green roofs. By comparison, the relationship-centric approach was much more present in interviews with NbS located in the Americas (in our case, mostly in Latin America and the Caribbean), also relating mostly to urban forests (see Tables S8 and S9 for full regional and type distributions).

Several interconnections between other themes also emerge from the co-occurrence matrix. On the general connection between *adaptation success* and *whose knowledge* was relevant to recognise it, *local* knowledge was the dominant form of knowledge that was deemed as being more useful to identify whether the NbS was *thriving* overall. Recognising whether NbS were *thriving* by using local knowledge mostly related to recognising *synergy* among different collaborating actors in projects as well as *sensitivity to the local context*, compared to other themes on *thriving*. The role of *technical* knowledge was then emphasised most often in terms of helping to understand the long-term *sustainability* of projects. *Scientific* knowledge was framed as playing only a modest role, mostly towards recognising *synergy* and *sustainability*.

The belief in a *hierarchy* of forms of knowledge co-occurred more often with some forms of knowledge than others. Those informants that highlighted the importance of *scientific* forms of knowledge also expressed a belief in a *hierarchy* among forms of knowledge, where *scientific* knowledge was held to be superior to others. By contrast, those highlighting the role of *local* knowledge more often spoke of the *pluralistic* nature of knowledge for adaptation where such hierarchies did not exist. That being said, *local* knowledge also often co-occurred with a discussion of the importance of *scientific* knowledge, though in a way that framed the two forms of knowledge as complementary rather than in competition.

The framing of adaptation as being *interconnected* among human and natural systems connected with the need for information about both *human* and *natural* systems. *Local* knowledge was framed as being key in understanding both *human* benefits in isolation and the interconnection of *human* and *natural* systems, whereas *scientific* knowledge was more strongly associated with understanding either *human* or *natural* systems separately.

Summarising these results, several components of *ways of being* and *ways of knowing* urban adaptation through NbS were identified through thematic analysis. Themes on *ways of being* included different definitions on what adaptation is (*hazard-centric or relationship-centric*), how *successful adaptation* is defined, as well as how the *connectivity* between human and natural systems was framed in the context of adaptation goals. Themes on w*ays of knowing* then differentiated between *for whom* and *from whom* knowledge was needed to define and evaluate adaptation goals, as well as whether a *hierarchy* existed among these forms of knowledge. From the co-occurrence of these different themes on *ways of being* and *knowing*, two distinct *ways of doing* urban adaptation through urban NbS were identified that were also statistically significant, which were called the *hazard-centric* and *relationship-centric* approaches. Though some differences between these approaches were slight in terms of their relationship with other themes, the two groups differed mostly in terms of their understanding of adaptation success (*creating synergy*), from and for whom knowledge should be generated (*local* vs *technical* knowledge and *human* vs *both human and natural* systems), and knowledge *pluralism*.

## Discussion

Understanding what it means to be adapted to climate change (*ways of being*) and how we know adaptation is happening (*ways of knowing*) helps to distinguish two very different approaches to how adaptation is done (*ways of doing*). In the sections that follow, we chart how these different approaches to adaptation that clearly manifested in the interview data bring important insights to communities of research and practice on urban adaptation that call for a change in the way adaptation is understood and done. This change is located in the literature that calls for a “relational turn” in sustainability science that can be connected directly to urban adaptation through our results (West et al. 2020).

### A call for a relational turn in how adaptation is understood and done

Our results suggest that urban adaptation to climate change is not only about protecting urban dwellers from the risks posed by a changing climate but further deepening relationships among one another and with nature. Defining adaptation in this way, as well as assessing progress towards adaptation goals, requires knowledge plurality that recognises that human and natural systems are interconnected and inseparable. However, current high-level definitions of adaptation such as that of the IPCC do not refer to the role that these relationships and knowledge pluralism play in how adaptation is successfully done. Instead, these definitions explicitly separate adaptation in “human” and “natural” systems. Hence, here we attempt to re-imagine existing definitions of adaptation with a relational entry point that foregrounds interconnectivity as follows: *“Adaptation is the process of cultivating and strengthening relationships both among humans and between humans and nature in a way that respects their innate interconnectivity. These relationships help identify, motivate and guide necessary adjustments to actual or expected climate and its effects in order to moderate harm and maximise the potential for those relationships to thrive.”*

Rather than attempting to conclusively redefine core concepts of adaptation, we instead provide a re-imagination of the IPCC definition that maintains its core concepts and complements it with elements of relationality and interconnectivity that are supported by our data (Verlie 2019; Iossifidis and Garforth 2022). This re-imagined definition identifies the unique contributions of a relational entry point to adaptation compared to the *hazard-centric* approach. For example, our thematic analysis has generated several unique components of *thriving* that were more strongly associated with the *relationship-centric* approach that open new avenues of exploration to better identify how they can complement future research and practice. Complementing existing definitions of adaptation in this way could form part of a larger “relational turn” in adaptation research and practice that has already begun in other areas of sustainability science (West et al. 2020).

This relational turn draws attention to four key contributions of relationality to sustainability science: continuously unfolding processes, embodied experiences, reconstructing language and concepts, and ethics and practices of care (West et al. 2020). Some of these points have already made their way into how adaptation is currently defined (Table 3). For example, West et al. (2020) discuss how relationality calls particular attention to the importance of understanding sustainability challenges and their solutions as processes that are continually unfolding, rather than being static (Hertz et al. 2020).

**Table 3:** Comparison of IPCC and relational approach to defining adaptation in terms of four key elements of the “relational turn” according to West et al. (2020)

	IPCC definition	Relational approach
*Continuously unfolding processes*	Adaptation is defined as a process	Maintains process-oriented language
*Embodied experiences*	Embodied experiences are not located in the context of adaptation	Embodied experiences of thriving within relationships with others as well as nature are located in the context of adaptation
*Reconstructing language and concepts*	The end goal of adaptation is limited to adjusting to harm and exploiting opportunities	The language is expanded beyond adjustment and situates humanity as interconnected to the world around it. The language of adjustment is maintained but re-contextualised in terms of how relationality may help “identify, motivate, and guide” action taken to adjust to a changing climate
*Ethics and practices of care*	Not mentioned	Relational framing invokes ethics and practices of care as adaptation is something that is done in the context of cultivating and strengthening relationships for shared thriving

Adopting a relational approach to adaptation clarifies the manner in which language and concepts actively shape reality, rather than merely reflecting it (Cook and Wagenaar 2012). Viewed through this lens, it is possible to better identify how definitions of adaptation serve to limit its scope to “*adjusting…* to *moderate* harm or *exploit* beneficial opportunities,” where dimensions of this process among human and natural systems are isolated by providing separate definitions of adaptation in human and natural systems. A re-imagined perspective would expand adaptation to include notions of thriving under different climatic conditions in a way that respects the interconnection both among humans and the world around them, beyond simply adjusting to change (Abson et al. 2017; Artmann 2023).

Re-framing adaptation with the language of relationality and interconnectivity centres adaptation within ethics and practices of care, capturing the embodied experiences of thriving within those relationships. It does so by calling attention to what is argued to be the root cause of, and solution to, issues posed by climate change, including adaptation: mutually destructive relationships among humans and between humans and the “more than human” world (Haverkamp 2021). Others have highlighted how defining adaptation relationally has the potential to embed ethics and practices of care into a definition of adaptation as it makes explicit the role and power of emotion, empathy, and connectedness as levers for positive change already highlighted across numerous sustainability fields (Nightingale 2016; Jax et al. 2018), including in the realm of adaptation (Nightingale et al. 2022; Riechers et al. 2022, 2021). Relational approaches can also provide an opportunity to address other challenges at the core of defining and practising adaptation, for example, unequal distribution of power and agency among actors (Garcia and Tschakert 2022), as well as the need for intersectionality in understanding climate vulnerability (i.e. overlapping layers of disadvantage or privilege along lines of racial identity, social or economic class, gender identity, ability, sexual orientation, among others) (Amorim-Maia et al. 2022).

Our re-imagination of adaptation pushes beyond the existing literature focusing on the heuristics of adaptation discussed in earlier sections by providing alternative starting points to understanding the concept (Preston et al. 2015; Vogel and Henstra 2015; Nalau et al. 2021). Our approach builds on an emerging body of the literature that incorporates these concepts into transformative governance and learning for urban climate change adaptation through NbS (Wickenberg et al. 2022) as well as adaptation governance (Marion Suiseeya et al. 2021; Sebastian and Jacobs 2021; Burger et al. 2023) and planning (Dujardin 2020). Integrating relationality into adaptation is rooted in Indigenous and feminist fields of research (Haverkamp 2021; Johnson et al. 2022), as well as empirical work conducted across numerous regions in the Global South (Kuruppu 2009; Alare et al. 2022; Chakraborty et al. 2023; Mubai et al. 2023).

### Implications of the relational turn in adaptation

Currently hazard-centric MERL practices prioritise evaluation of results of implementation (outputs) rather than longer-term outcomes and impacts (Goonesekera and Olazabal 2022; Oakes et al. 2022; Chmutina et al. 2023). Our findings echo previous calls to refocus MERL processes instead on evaluating whether or not local needs are being met rather than just outputs alone (Caillon et al. 2017; Dilling et al. 2019). Further study is then needed on how the kind of synergetic relationships highlighted by our informants as critical to adaptation success can emerge and how they influence processes and outcomes within adaptation interventions. On a practical level, awareness of the influence of relationships and networks needs closer attention, particularly for local practitioners responsible for adaptation processes to understand their role in creating networks and communities of trust through their adaptation projects. Similar calls have been made in the recent literature, focusing, for example, on identifying and supporting individuals within projects that operate as bridges between different actors and forms of knowledge within adaptation projects through connective leadership (Oakes et al. 2022; Peterson St-Laurent et al. 2022). Further study and practice is needed to uncover what kind of relationships we need to make adaptation work and how they can be identified and fostered, rather than identifying specific indicators and metrics alone.

Our results also imply that knowledge plurality under a relational approach to adaptation requires that scientific knowledge takes its place among other forms of knowledge that are equally valid, legitimate, and useful. Scientists need to be humble in recognising that their way of knowing is one of many (West et al. 2020; West et al. 2021). This change in positioning will require some scientists to challenge their belief in a hierarchy of knowledge (Eriksen et al. 2015; Bamzai-Dodson et al. 2023). Doing relational adaptation requires relational science, which must acknowledge that science too “results from relationships among human societies, scientists and the subject under study” (Eyster 2023).

Addressing the challenges of a relational turn in adaptation requires overcoming paradoxes that future research and practice must tackle. Discussing relationality within political processes, such as by eliciting urban climate imaginaries for adaptation planning, demands high engagement from diverse actors to ensure credibility and equity. However, this engagement also complicates the creation of shared visions as they become more diffuse and may also reveal disagreements about desirable futures (West et al. 2020). A key challenge is to provide practical guidance on how these imaginaries can be incorporated into adaptation planning across governance levels in ways that are inclusive yet decisive. Progress is already being made, such as using normative future visioning tools to enhance local monitoring, evaluation, and learning processes (Pelling et al. 2024).

### Limitations of our findings

Our results should be interpreted in light of several methodological limitations, specifically the sample of interviews. Although sufficient to form the basis of a thematic analysis, the sample of 15 interviews may limit the generalisability of our results. However, we have noted how our aim is not to generalise, but to particularise the diversity of adaptation imaginaries within a heterogeneous sample of NbS practitioners.

Several other aspects of the data may inform how they should be interpreted, for example, that they come from different types of NbS, respond to different hazards, and come from different organisational types. For example, a majority of the sample related to urban greening in some way (relevant to terrestrial ecosystems, for example urban forests, green roofs, or urban agriculture) which may mean that our results relate more heavily to adaptation projects relating to these ecosystems or require different kinds of technical expertise. Further, a majority of informants were from non-governmental actors implementing NbS (i.e. either international organisations or grassroots movements) which may further influence the kinds of themes that emerged based on their specific organisational goals or priorities.

The relatively small sample size may also mean that the thematic analysis was sensitive to different heterogeneous attributes of projects, for example that projects were developed on the basis of different criteria for project funding, national regulations and priorities, available/relevant knowledge and solutions, as well as local culture (including both the general culture of different societies and planning and governance cultures). Addressing this sensitivity through the mixed method approach taken was therefore crucial to be able to quantify and identify which sub-themes were more influential in differentiating the two identified approaches to adaptation, given that the numerical difference between the distribution of some sub-themes between the approaches was slight.

## Conclusions

We have approached the question of whether and how real-world experiences with applying NbS to urban adaptation challenges could enrich how adaptation goals are formulated, and what information is required to assess progress towards these goals. Our answers to these questions were drawn from the on-the-ground perspectives of those involved in implementing urban NbS. The themes emerging from the analysis of these perspectives suggest that in addition to the current hazard-centric framing of adaptation and evaluation metrics, relationality and interconnectivity were also important concepts. Drawing on the literature that has tracked a relational turn in sustainability sciences more generally helped us to analyse how we could re-imagine a definition of adaptation that accounted for relationality and interconnectivity. We have argued that this definition, while not replacing existing ones, can help access deeper leverage points for urban transformation through adaptation strategies that address not only climatic hazards, but also the unbalanced relations of power, agency, and intersectional vulnerability that create and reinforce them.

Moving forward, our main recommendation is to clarify and expand currently limited definitions of adaptation at the institutional level. This change in entry point to adaptation may help expose possible root causes of adaptation challenges, and illuminate ways of addressing them that align with local needs and priorities as well as interconnected biodiversity conservation and social justice challenges. While we have forwarded a single definition of adaptation here, decision-makers on NbS to adaptation ought to invest more time, energy, and resources into similarly defining what adaptation means to those they intend to support in a non-hierarchical and pluralistic manner. In our case, we have highlighted the role of relationships and interconnectivity in this discussion; however, we acknowledge that additional or alternative imaginings of adaptation are equally possible and indeed required to reflect local contexts.

Our findings open several avenues for further research in the field of urban adaptation more broadly. For example, critics of the “relational turn” in sustainability science question the applicability of the concept to real-world adaptation practices (Raymond, 2021). While we have highlighted the specific ways in which doing so could contribute to adaptation in the practical sense, future research is nonetheless required to understand how relational understandings of adaptation contribute to real-life MERL practices for urban NbS. Applied to adaptation more broadly, this line of research would also help clarify whether relationality and interconnectivity are a unique feature of adaptation through NbS, or whether it applies to adaptation more broadly. Clarifying the significance of the relational turn in this way may enable future research to explore in what other ways local imaginaries can enrich definitions of adaptation and information used to assess progress towards adaptation goals. Our results make a first step in this direction by identifying the potential for local knowledge to challenge and reshape accepted wisdom on what adaptation is and how we can know it is happening.

## Supplementary Information

Below is the link to the electronic supplementary material.

Supplementary file1 (PDF 501 KB)

## Footnotes

1 As we only discuss NbS in the context of urban adaptation benefits, the use of the term NbS throughout this paper should be read to refer only to that context.
