# Supplementary material for: A relational turn in climate change adaptation: Evidence from urban nature-based solutions
Source: Ambio. 2024 Dec 7;54(3):520–35. doi: 10.1007/s13280-024-02090-9 (PMC11780061; doi:10.1007/s13280-024-02090-9)
Supplement: Supplementary file 1 — Supplementary file1 (PDF 501 KB) [file 13280_2024_2090_MOESM1_ESM.pdf]

Ambio
Supplementary Information
This supplementary information has not been peer-reviewed.

Title: **A relational turn in climate change adaptation: evidence from urban nature-based solutions**

Sample description

The below Table S1 describes the interview sample used. As much information about the nature-based solutions (NbS) and Informants is given to ensure the privacy and comfort of participating in the interviews. In some cases, Informants expressed an unwillingness to disclose more personal information, and to ensure that information provided is comparable across the sample, only that information that could be generally disclosed across Informants is given.

Table S1. Descriptions of each Informant in terms of the location (city/country/region), NbS type, organisation type, as well as the interview length in minutes and pages of transcripts taken. Cells are left blank where Informants did not wish to disclose this information. Typologies for NbS and actor type are adapted from a previous study (Goodwin et al., 2023) and the self-identification of Informants.

ID #	City	Country	Region	NbS Type	Actor Type	Interview length (minutes)	Transcript length (pages)
1	Bogotá	Colombia	Americas	Urban forests	Grass-roots movement	57	7
2	Brasilia	Brazil	Americas	Urban forests	Grass-roots movement	90	13
3	Cairo	Egypt	Africa	Green roofs	International organisation	39	10
4	Cairo	Egypt	Africa	Green roofs	International organisation	48	9
5	Colombo	Sri Lanka	Asia	Urban forests/ wetlands	International organisation	39	9
6	Durban	South Africa	Africa	Urban forests	Municipal government	58	10
7	Glasgow	United Kingdom	Europe	Urban forests /river re-naturalisation	National government department	52	8

ID #	City	Country	Region	NbS Type	Actor Type	Interview length (minutes)	Transcript length (pages)
8	Hamburg	Germany	Europe	Green roofs	Municipal government	55	9
9	Jinhua	China	Asia	Urban river/ wetlands restoration	Research/ technical	49	6
10	Muyinga	Burundi	Africa	Urban forests	International organisation	43	7
11	Pune	India	Asia	Urban forests	Grass-roots movement	44	7
12	-	Brazil	Americas	Urban agriculture	International organisation	35	6
13	São Paulo	Brazil	Americas	Public park/garden	Grass-roots movement	53	8
14	São Paulo	Brazil	Americas	Urban forests	Grass-roots movement	73	8
15	-	Spain	Europe	-	Research/ technical	81	16
					Average	54	9
					Total	816 (~13 hours)	133

Sample criteria

In the main text of this paper, four sampling criteria are given:

1. the experience of the Informant with the development of the NbS, implementation, and involvement in monitoring and evaluation,
2. diversity of NbS type,
3. geographical diversity, and
4. availability and ability to take part in online interviews in English within the interview period (March to May 2023).

The Informants' experience was determined through prior contact with the project representatives. They were informed of the interview topic and questions and asked about their role (i.e. if they formed part of the core design/implementation team for the NbS). They were also asked if they would be able to answer the questions confidently and if not, to identify

a qualified representative. The diversity of NbS type within the interview sample refers to our intention to interview representatives across the different NbS types represented in the sub-sample as they were initially categorised by Goodwin et al. (2023), which were mostly wetlands (33% of the sub-sample), public parks/gardens (33%), green roofs (21%), urban forests (14%), and river re-naturalisation (10%), with a smaller representation of urban agriculture (1%). Geographical diversity then meant we also strove to interview representatives from NbS across the five primary world regions according to the United Nations categorisation (Table 1) (United Nations, 1999). A finer-grain representation of sub-regions was not possible given the limitations of the original sample in that most sub-regions were under- or unrepresented.

Other practical exclusion criteria included whether contact information could be feasibly found on the database entries themselves (e.g. if a name and email were provided), or through subsequent internet searches for responsible parties where the first ten results were analysed searching with the NbS name and city/country it was implemented within. Based on the availability of contact information and the resources available, we aimed to interview representatives from 3-4 cities across each world region (excluding Oceania, given that there was only one Oceanian project within the sub-sample). Because of the relatively higher number of European cities within the sample, this also meant that fewer were contacted to allow for a balance across world regions (Table 1). While in most cases almost all NbS were contacted, this was not the case in Europe. To fit without our intended sample size, additional criteria were needed to choose among the NbS in European cities. The selected European NbS were prioritised because of their transformative potential determined from the previous work that the sample was drawn from (Goodwin et al., 2023), therefore evidencing an above-average level of engagement in processes relevant to the interview questions (e.g., theory of change, and more thorough MERL processes). In total, 30% of the representatives identified did not respond, 28% of database entries did not contain identifiable contact information, and 23% were not contacted.

Table S2. Results of the sampling procedure per world region. “Interviewed” refers to situations where email contact was established, and interviews were successfully conducted. In one case, an interview was scheduled but the Informant did not attend for unknown reasons (“Interview not attended”). Where email contact was made but no response was received these were categorised as “No response”. In all cases, follow-ups were sent after a week had passed. “Contact information not found” refers to situations where no information could be found online to contact a project representative. “Not contacted” refers to those who may have had contact information, but were not contacted.

Region	Count	% Total sample
**Africa**	14	19%
Interviewed	2	3%
Interview not attended	1	1%
No response	6	8%
Contact information not found	4	5%
Not contacted	2	3%
**Americas**	16	22%
Interviewed	5	7%
No response	10	14%
Contact information not found	1	1%
Not contacted	0	0%
**Asia**	8	11%
Interviewed	3	4%
No response	3	4%
Contact information not found	1	1%
Not contacted	1	1%
**Europe**	35	47%
Interviewed	3	4%
No response	3	4%
Contact information not found	15	20%
Not contacted	14	19%
**Oceania**	1	1%
Interviewed	0	0%
No response	1	1%
Contact information not found	0	0%
Not contacted	0	0%
**Total**	**74**	**100%**

Sample size

The sufficiency of a sample size to adequately describe themes within a thematic analysis is controversial, without a defined number. Some argue that a sample size between 6 and 16 interviews (as in our case) would be sufficient. In taking this as a general rule of thumb in addition to the above pragmatic limitations of the sampling process (i.e., inability to reach the entire sample, lack of response, balancing representation across the data in terms of world region and NbS type, and resources), we additionally adopt the approach of Braun and Clark (2021) who describe five (5) pragmatic considerations that should be disclosed regarding sampling decisions made for thematic analyses that invite the reader to understand the reflexive nature of the study design (Table S3).

Table S3. Summary of the five reflexive considerations to be made when evaluating the sufficiency of a sample size for thematic analysis (Braun and Clarke, 2021).

**Consideration**	**Response**
Sample sufficiency and thematic saturation understanding	Six to sixteen interviews (though only a “rule of thumb”).
Type of thematic analysis conducted	Following the cited approach of Boyatzis.
Paradigmatic, ontological, and epistemological assumptions underpinning research	Constructivist account of research where knowledge and its meaning are not “discovered” as it “resides at the intersection of the data and the researcher’s contextual and theoretically embedded interpretative practices”.
Definitions of codes and themes	Themes are created as codes when they play an influential role in explaining the Informants' answer to the questions asked, and the research question. These are given in a code book in the main text of this paper, including a detailed description as well as direct quotes.
Justification of numeric criteria	Numeric criteria are not sufficient alone to describe the sufficiency of the sample. The sufficiency of the sample is more a function of its adequacy to answer the research question, judged by the richness and complexity of the responses given within the sample, as well as pragmatic limitations placed on the sampling procedure that are described above and in the main text.

We have already addressed our understanding of sample sufficiency and the type of thematic analysis we conducted in the main paper, as well as our approach to defining codes and

themes. In this Supplementary Information, we add to this by reflecting on how our conceptualisation of thematic sufficiency or saturation relates more to the reflexive process of interpretation of the interviews conducted that aims to summarise (and be faithful to) the situated worldviews of Informants rather than representative or generalisable of NbS or adaptation as a whole. As our research is exploratory and exists in a yet under-developed research niche without a wealth of empirical knowledge to draw from, we do not seek to generalise our results beyond the interview sample, and further acknowledge the unfitness of our sample for this task while inviting future work to test and challenging the theory and hypotheses we present here. This then reflects our own paradigmatic, ontological, and epistemological assumption underpinning our research that understands research to be "a situated, reflexive and theoretically embedded practice of knowledge generation or construction, rather than discovery" (Braun and Clarke, 2021). This does not absolve us from justifying our sample size but rather, on the contrary, describing our reflexive process of developing the sampling procedures, as is provided here.

Interview structure

Semi-structured Interviews were originally done to discuss the process of selection and prioritisation of information used to evaluate project outcomes. However, an in-depth discussion of project goals emerged from those interviews, including how they were identified, and what and whose knowledge was needed to assess progress towards them.

Two guiding questions were identified to facilitate discussion on the process of selection and prioritisation of indicators: (1) what was the process for selecting the indicators chosen, and (2) do you feel these indicators helped to measure progress towards your goals? Follow-up questions were also asked to determine if a key topic had been missed, or whether someone else should be spoken with to understand the themes arising from the interview as a way of snowballing. Only one Informant identified another person who needed to be consulted to complement their answers, and so in total 15 Informants were interviewed across 14 projects. Several probes were used below to facilitate the discussion to ensure the conversation flowed (Table S4).

Table S4. A list of questions asked to interviewees, along with a selection of probes that could be used to focus discussions.

Question	Text	Suggested probes
Question 1	What was the process for selecting the indicators you chose?	● Who was involved and what were their roles?<br>○ Where are you within this process in terms of your role/responsibilities?<br>○ How central were you to decision-making?<br>○ Was everyone involved who should have been involved?<br>● How did you prioritise among different indicators?<br>○ What features of the indicators you chose made them "good" indicators to prioritise?<br>● How did you connect immediate results to medium and long-term project goals?<br>● What did you aim to achieve using the selected indicators (i.e., what were your goals)?<br>○ What were your motivations for achieving these goals?<br>○ Are these motivations based on your own experience or something required by the project?<br>■ Which experiences do you think helped you develop these motivations?
Question 2	Do you feel these indicators helped you measure progress towards your project goals?	● Were you able to ensure that the indicators you used included all relevant information and had all the key features you identified as "good" indicators?<br>○ What was key to your success in achieving your goals, or what challenges did you face in achieving them?<br>■ How were these challenges overcome / what would you have needed to overcome them?<br>● How was the process once the indicators were selected and implemented?

Questions, and associated probes, were framed using open-ended language that aimed to trigger a discussion on the Informant´s point of view and how that influenced their work designing and implementing the NbS (e.g., "How do you…", "In your experience…"), rather than the official project narrative that could be otherwise obtained through a document analysis.

Reliability testing

To ensure the reliability of the coding process, we employed memos to document and reflect on each methodological step outlined in the main paper. These memos served as a crucial tool in maintaining the reliability and validity of our findings, addressing a key challenge inherent in interpretive processes such as inductive coding of qualitative data (Barbour, 2014; King and Horrocks, 2010, pp. 164–165). For instance, utilising memos to reflect on the coding process is a common technique aimed at mitigating researcher biases and safeguarding against premature theorisation. This practice keeps the analysis transparent, preventing potential contamination of the findings.

Because of the inductive nature of the data analysis, further inter- or intra-coder reliability of the coding process was not deemed appropriate (Barbour, 2014). Instead, in addition to reflective journaling and memo-keeping, "thick descriptions" of the themes analysed are provided in the main text to provide examples to the reader on how the themes analysed connected to the direct words of the Informants supported by specific quotes (O'Connor and Joffe, 2020).

In addition to memo keeping, additional testing was performed to ensure the reliability of codes developed through the thematic analysis in two phases. The first phase occurred once the initial codebook was developed. The initial set of codes was tested on a purposive sample of 3 interviews, chosen because of their thematic diversity, to validate the codes and ensure their applicability and relevance to the entire interview dataset. Following any initial adjustments made, the codes were then applied to the full dataset. The second phase then occurred once the coding of all interviews was completed. This phase involved checking and re-examining all coding units (passages of text, separated by topic of conversation) that were coded to each theme to ensure consistency.

Co-occurrence matrices

Table S5 below displays the co-occurrence matrix that was the basis of the scoring and clustering process described in the main paper. Then, an abbreviated co-occurrence matrix is given that focuses on the differences between the aggregated hazard- and relationship-centric approaches (Table S6). The results of the additional statistical tests performed are then summarised (Table S7).

Table S5. Co-occurrence matrix of all themes from the thematic analysis, where the values given represent the number of times the themes in the coinciding rows and columns co-occurred in a single coding unit (passage of text with a single topic).

	Theme	Sub-Themes	Reducing impact of climate hazards	Supporting adaptive capacity	Relationships among people	Relationships with nature	Relationships among people and with nature	Separate	Interconnected	Sustainability	Context sensitivity	Flexibility	Synergy	Growing	Hierarchical	Non-hierarchical	Scientific	Indigenous	Local	Technical	Human	Non-human	Human and non-human
Ways of being	Hazard-centric	*Reducing impact of climate hazards*		1	0	0	0	2	8	2	1	0	1	2	1	0	5	0	9	15	23	4	11
		Supporting adaptive capacity	1		5	0	0	0	1	3	0	0	1	0	0	0	1	0	13	6	20	0	1
	Relationship-centric	*Relationships among people*	0	5		0	0	0	1	2	4	1	16	2	0	7	4	1	27	0	9	0	6
		Relationships with nature	0	0	0		0	0	8	0	2	0	1	0	0	1	3	2	11	1	4	2	8
		Relationships among people and with nature	0	0	0	0		0	3	1	0	0	1	2	0	2	1	0	6	1	1	0	6
	Connectivity	*Separate*	2	0	0	0	0		0	0	0	0	0	0	0	0	0	0	1	1	1	1	1
		Interconnected	8	1	1	8	3	0		1	3	1	2	5	1	5	6	2	16	3	7	0	22
	Adaptation success	*Sustainability*	2	3	2	0	1	0	1		0	0	1	1	0	0	5	0	11	10	8	2	1
		Context sensitivity	1	0	4	2	0	0	3	0		2	2	0	0	2	2	0	10	5	4	2	4
		Flexibility	0	0	1	0	0	0	1	0	2		0	1	0	1	3	0	5	2	0	0	2
		Synergy	1	1	16	1	1	0	2	1	2	0		2	1	8	8	1	23	6	7	1	3
		Growing	2	0	2	0	2	0	5	1	0	1	2		0	4	3	0	7	2	3	1	5
Ways of knowing	Knowledge hierarchy	*Hierarchy*	1	0	0	0	0	0	1	0	0	0	1	0		0	7	0	1	4	1	2	2
		Pluralism	0	0	7	1	2	0	5	0	2	1	8	4	0		2	1	18	2	3	0	5
	Knowledge from	*Scientific*	5	1	4	3	1	0	6	5	2	3	8	3	7	2		1	16	10	20	15	11
		Indigenous	0	0	1	2	0	0	2	0	0	0	1	0	0	1	1		1	0	0	0	1
		Local	9	13	27	11	6	1	16	11	10	5	23	7	1	18	16	1		6	34	9	21
		Technical	15	6	0	1	1	1	3	10	5	2	6	2	4	2	10	0	6		21	16	12
	Knowledge for	*Human systems*	23	20	9	4	1	1	7	8	4	0	7	3	1	3	20	0	34	21		0	0
		Natural systems	4	0	0	2	0	1	0	2	2	0	1	1	2	0	15	0	9	16	0		0
		Human and natural systems	11	1	6	8	6	1	22	1	4	2	3	5	2	5	11	1	21	12	0	0	

Table S6. Co-occurrence matrix of the aggregated hazard-centric and relationship-centric themes with all other themes, where the values given represent the number of times the themes in the coinciding rows and columns co-occurred in a single coding unit (passage of text with a single topic).

	**Themes**	**Sub-themes**	**Hazard-centric**	**Relationship-centric**
**Ways of being**	**Connectivity**	*Separate*	2	0
		Interconnected	9	12
	**Adaptation success**	*Sustainability*	2	1
		Context sensitivity	1	5
		Flexibility	0	1
		Synergy	1	17
		Growing	2	4
**Ways of knowing**	**Knowledge for**	*Human and natural systems jointly*	13	20
		Natural systems	3	3
		Human systems	39	10
	**Knowledge from**	*Technical*	19	2
		Indigenous	0	3

		Local	15	38
		Scientific	5	7
	**Knowledge hierarchy**	*Hierarchy*	1	0
		Pluralism	0	10

Table S7. Summary of group effects between coding units that were coded as either hazard-centric or relationship-centric. These values represent the probability of a coding unit being coded as either approach in addition to other codes on ways of being and knowing. The higher of the two probabilities is underlined per sub-theme, with the highest difference in italics.

Guiding theory	Themes and sub-themes	Hazard-centric	Relationship-centric	Difference
Ways of being	**Connectivity**			
	Connected	*47%*	*53%*	*6%*
	Separate	52%	48%	4%
	**Adaptation success**			
	Context sensitivity	47%	53%	6%
	Flexibility	49%	51%	2%
	Sustainability	51%	49%	2%
	Synergy	*36%*	*64%*	*28%*
	Growth	48%	52%	4%
Ways of knowing	**Knowledge from**			
	Local	*31%*	*69%*	*38%*
	Scientific	48%	52%	4%
	Technical	64%	36%	28%
	Indigenous	47%	53%	6%
	**Knowledge for**			
	Human systems	*75%*	*25%*	*50%*
	Natural systems	50%	50%	0%
	Both	44%	56%	12%
	**Knowledge hierarchy**			
	Hierarchy	51%	49%	2%
	Pluralism	*42%*	*58%*	*16%*

Table S8. Summary of the percentage of coding units per region.

Region	Hazard-centric	Relationship-centric
Americas	18%	77.99%
Africa	45.29%	14.49%
Asia	18.36%	5.39%
Europe	18.36%	2.13%

Table S9. Summary of the percentage of coding units per NbS type.

Type	Hazard-centric	Relationship-centric
Urban forests	35%	86%
Urban agriculture	5%	0%
Green roofs	31%	11%
River re-naturalisation	13%	0%
Public park/garden	1%	3%
Wetlands	14%	0%

References in Supplementary Information

Barbour, R., 2014. Quality of Data Analysis, in: Flick, U. (Ed.), The SAGE Handbook of Qualitative Data Analysis. SAGE Publications, Inc., 1 Oliver's Yard, 55 City Road London EC1Y 1SP. https://doi.org/10.4135/9781446282243

Boyatzis, R.E., 2010. Transforming qualitative information: thematic analysis and code development, Nachdr. ed. Sage, Thousand Oaks, Calif.

Braun, V., Clarke, V., 2021. To saturate or not to saturate? Questioning data saturation as a useful concept for thematic analysis and sample-size rationales. Qual. Res. Sport Exerc. Health 13, 201–216. https://doi.org/10.1080/2159676X.2019.1704846

Goodwin, S., Olazabal, M., Castro, A.J., Pascual, U., 2023. Global mapping of urban nature-based solutions for climate change adaptation. Nat. Sustain. 6, 458–469. https://doi.org/10.1038/s41893-022-01036-x

King, N., Horrocks, C., 2010. Interviews in qualitative research. SAGE, Los Angeles.

O'Connor, C., Joffe, H., 2020. Intercoder Reliability in Qualitative Research: Debates and Practical Guidelines. Int. J. Qual. Methods 19, 160940691989922. https://doi.org/10.1177/1609406919899220

United Nations. Standard country or area codes for statistical use (M49) [Internet]. 1999. Available from: http://unstats.un.org/unsd/methods/m49/m49.htm
